# Acute kidney injury during daptomycin versus vancomycin treatment in cardiovascular critically ill patients: a propensity score matched analysis

**DOI:** 10.1186/s12879-019-4077-1

**Published:** 2019-05-20

**Authors:** Philippe Gaudard, Marine Saour, David Morquin, Hélène David, Jacob Eliet, Maxime Villiet, Jean-Pierre Daures, Pascal Colson

**Affiliations:** 1 PhyMedExp, University of Montpellier, CNRS, INSERM, Department of cardiothoracic Anaesthesiology and Critical Care Medicine, CHU Montpellier, Montpellier, France; 2 Department of cardiothoracic Anaesthesiology and Critical Care Medicine, CHU Montpellier, Montpellier, France; 3 Infectious and Tropical Diseases Department, CHU Montpellier, Montpellier, France; 4 Clinical Pharmacy Department, CHU Montpellier, Montpellier, France; 5 Laboratory of Biostatistics and Epidemiology EA2415, University Institute for Clinical Research, Montpellier, France

**Keywords:** Vancomycin, Daptomycin, Acute kidney injury, Nephrotoxicity, Infective endocarditis, Foreign body associated infection, Cardiovascular surgery

## Abstract

**Background:**

Gram-positive organisms are a leading cause of infection in cardiovascular surgery. Furthermore, these patients have a high risk of developing postoperative renal failure in intensive care unit (ICU). Some antibiotic drugs are known to impair renal function. The aim of the study was to evaluate whether patients treated for Gram-positive cardiovascular infection with daptomycin (DAP) experienced a lower incidence of acute kidney injury (AKI) when compared to patients treated with vancomycin (VAN), with comparable efficacy.

**Methods:**

ICU patients who received either DAP or VAN, prior to or after cardiovascular surgery or mechanical circulatory support, from January 2010 to December 2012, were included in this observational retrospective cohort study. We excluded patients with end stage renal disease and antibiotic prophylaxis. The primary endpoint was the incidence of AKI within the first week of treatment. Secondary endpoints were the incidence of AKI within the first 14 days of treatment, the severity of AKI including renal replacement therapy (RRT), the rates of clinical failure (unsuccessful infection treatment) and of premature discontinuation and mortality. To minimize selection bias, we used a propensity score to compare the 2 groups. Univariate and multivariate analysis were performed to determine factors associated with AKI.

**Results:**

Seventy two patients, treated for infective endocarditis, cardiovascular foreign body infection, or surgical site infection were included (DAP, *n* = 28 and VAN, *n* = 44). AKI at day 7 was observed in 28 (64%) versus 6 (21%) of the VAN and DAP patients, respectively (*p* = 0.001). In the multivariate analysis adjusted to the propensity score, vancomycin treatment was the only factor associated with AKI (Odds Ratio 4.42; 95% CI: 1.39–15.34; *p* = 0.014). RRT was required for 2 (7%) DAP patients and 13 (30%) VAN patients, *p* = 0.035. Premature discontinuation and clinical failure occurred more frequently in VAN group than in DAP group (25% versus 4%, *p* = 0.022 and 42% versus 12%, respectively, *p* = 0.027).

**Conclusions:**

Daptomycin appears to be safer than vancomycin in terms of AKI risk in ICU patients treated for cardiovascular procedure-related infection. Daptomycin could be considered as a first line treatment to prevent AKI in high-risk patients.

## Background

Gram-positive organisms are a leading cause of cardiac or vascular infection, particularly in patients undergoing cardiovascular surgery with foreign body implantation. These infections may be related to the surgery indication (i.e. infectious endocarditis) or to postoperative infectious complication (i.e. surgical site infection, cardiovascular device or prosthesis infection). Patients with sepsis or septic shock require an appropriate and adequate antibiotic regimen, including early administration, broad spectrum and high doses [1, 2]. The antibiotic treatment is started often probabilistically, then adapted to isolated strains.

Acute kidney injury (AKI) is a frequent complication that occurs in 15 to 25% of patients after vascular surgery [3–6], and up to 40% of patients after cardiac surgery [7–9]. In this surgical population, AKI that requires renal replacement therapy (RRT) ranges from 1 to 6% [8, 9]. AKI compromises seriously short and long-term prognosis of critically ill patients, namely patients admitted in intensive care unit (ICU) with at least one organ failure, especially during sepsis [10, 11]. Several AKI risk factors have been identified including a chronic pathology of the patient such as kidney failure or diabetes, acute kidney injury related to hemodynamic disorders during surgery, including cardiopulmonary bypass, or sepsis, and the use of nephrotoxic agents such as some antibiotics, colloids or iodine contrast agents [8, 9, 12, 13]. Avoiding nephrotoxic agents is therefore strongly recommended in ICU patients, to reduce the incidence of AKI, or to reduce its severity [14].

In this respect, Daptomycin, which showed good safety and efficacy in previous studies [15, 16], may be a safer alternative to vancomycin. In a first large validation study, non-inferiority of daptomycin for the treatment of bacteraemia and endocarditis caused by *Staphylococcus aureus* against standard treatment (low dose gentamycin plus vancomycin or penicillinase-resistant penicillin) was demonstrated with a lower rate of renal dysfunction [17]. However, the criteria used to define renal dysfunction was not the currently accepted definition for AKI issued by the Kidney Disease: Improving Global Outcomes (KDIGO) consortium [14]. Moreover, there is some controversy regarding vancomycin nephrotoxicity when used in continuous intravenous infusion [12, 18]. To our knowledge, no study has compared the nephrotoxicity of daptomycin and vancomycin in ICU patients yet.

The aim of this cohort study was to assess whether the use of daptomycin, was associated to a lower incidence of AKI than vancomycin in cardiovascular ICU patients, with similar efficacy.

## Methods

### Study design

This is a retrospective observational study with a propensity score adjustment to reduce the bias of selection for a comparative analysis between two antibacterial treatments used in routine care. The ethics committees (“CPP Sud Méditerranée IV”, N°Q-2015-05-03 and the “CCTIRS”, N°15.670) approved the study. The requirement for informed patient consent was waived since the nature of the study was retrospective. The data collection was authorized by the “CNIL” (N°DR-2015 − 643).

### Targeted population

From January 2010 to December 2012, patients who were admitted to a cardiothoracic and vascular surgical ICU and who received either daptomycin (DAP group) or vancomycin (VAN group), identified from the delivery drug list of the hospital pharmacy, were screened for analysis.

The following criteria were required for inclusion: (i) Patient older than 18 years; (ii) Suspected or proven cardiac, vascular or profound surgical site infection with Gram-positive cocci (GPC) methicillin-resistant (MR) strains (including probabilistic treatment for patients with acquisition of MR risk factors); (iii) Treatment duration greater than or equal to 48 h (at least 2 doses of daptomycin administered or 2 days of vancomycin infusion); (iv) Antibiotic treatment started in peri-operative (from 48 h before the onset of surgery) or in postoperative period (during ICU stay). Patients with prophylaxis indication of antibiotics, kidney disease on chronic dialysis or acute onset of RRT before initiation of DAP or VAN treatment, or staphylococcus pneumonia were excluded.

### Interventions

The indication for a probabilistic or documented treatment with broad spectrum antibiotics against GPC followed institutional protocols and national recommendations for the specific population of patients admitted to ICU with sepsis or septic shock in the context of cardiovascular surgery or cardiogenic shock. Infectious endocarditis means infection on native or prosthetic valve or pacemaker leads. Surgical site infection is an infection located on the cutdown tissue or profound structures, i.e. sternal infection or mediastinitis. Ventricular assist device (VAD) provides a long-term mechanical circulatory support and only profound device-related infections occurring during the postoperative period of the implantation were considered in this study (excluding driveline or delayed infections). Vascular graft infection requires usually a probabilistic treatment after surgical samples during graft replacement, which is a complex and haemorrhagic surgery. Catheter related infections were identified by bacteraemia and positive culture of the removed catheter with the same strain and were aggressively treated in a population with cardiac or vascular prosthetic materials. The other conditions to treat were an anterior mediastinitis or a septic shock in waiting of documentation. Cardiovascular foreign body infection was defined by an infection related to intra-cardiac devices or prothesis or vascular graft with artificial tissue with development of bacterial biofilm.

Daptomycin was administered at a dose of 8 mg/kg in thirty-minutes intravenous infusion every 24 h in patients without severe impairment of kidney function or every 48 h in case of GFR below 30 ml/min/m^2^. The creatine-kinase (CK) level was measured before the initiation of DAP and at least once a week to assess the occurrence of muscular toxicity defined by an increase of CK up to 3-fold the upper superior limit without any evidence of member ischaemia.

Vancomycin intravenous treatment was initiated by a loading dose of 30 mg/kg in 1 h and followed by a continuous maintenance infusion dosing between 15 and 30 mg/kg/d. The VAN dose was adapted to achieve a target serum vancomycin steady-state concentration of 20–30 mg/L assessed by a daily pharmacologic monitoring (therapeutic drug monitoring).

### Data collection

Demographic data, and main clinical characteristics were collected. Renal function was assessed by daily measurement of creatinine and urinary output monitoring in ICU. The need for RRT was left to the decision of the physician in charge of the patient, mainly because of threatening metabolic disorders (metabolic acidosis, hyperkalaemia) or fluid overload. The AKI stage was established during the first 7 days and 14 days after the initiation of the studied treatment.

The factors associated to renal function impairment before the antibiotic treatment were collected for the calculation of the propensity score: severity score as the Simplified Acute Physiology Score II (SAPS II) at ICU admission and the Sequential Organ Failure Assessment (SOFA) score at initiation of treatment, history of chronic renal failure (creatinine clearance below 50 ml/min), creatinine and AKI before treatment initiation (defined as baseline), circulatory shock related to sepsis or heart failure (defined as systolic arterial pressure below 90 mmHg and refractory to fluid challenge with evidence of end-organ hypoperfusion like oliguria, alteration of mental status or high plasma lactate), need of cardiopulmonary bypass for surgery (within 48 h around the antibiotic initiation), use of other nephrotoxic agents (iodine contrast, aminoglycoside, ciclosporin), documentation of GPC bacteraemia.

### Study endpoints

The primary endpoint was the incidence of AKI within the first week of treatment as defined by KDIGO consensus [14].

The secondary endpoints were chosen to better characterize AKI and describe the outcome. The incidence of AKI within the first 14 days of treatment and the maximal decrease of GFR (estimated by CKD-EPI formula) during treatment were also reported. Besides KDIGO classification, severe renal failure was estimated as stage 2 or 3 of the AKI or a reduction in glomerular filtration rate (GFR) of more than 50%, and from incidence and duration of RRT. Clinical treatment failure, defined by either persistent positive cultures, worsening of clinical status, death due to initial infection, or relapse after the end of treatment was assessed in case of documented GPC infection. Incidence of premature discontinuation of treatment was defined when treatment was stopped because of adverse event or clinical failure except death. Mortality rate was calculated at day 28 and month 6.

### Statistical analysis

In a previous study [17], the incidence of renal dysfunction was estimated at 12% with daptomycin and 35% with standard therapy. However, a higher incidence of renal failure was expected in ICU population, as much as twice the incidence observed in the reference study [10, 19]. Therefore, we assumed that AKI may occur in 60% VAN patients, meaning that a reduction to 20% in DAP group would be significant for a risk at 5% and a power equal to 85%, provided 52 patients (at least 26 in both groups) were included.

Qualitative variables were expressed in percentages and compared with the Chi-square test or Fisher’s exact test. Quantitative normally distributed variables were expressed as means and standard deviations and assessed with the Student’s t-test. Medians and 25th to 75th percentiles defined quantitative not normally distributed data, for which differences were analysed by the Wilcoxon-Mann-Whitney test. *P* values below the 0.05 level were considered to indicate statistical significance.

Since treatments were not randomized, we used the propensity score method for primary endpoint analysis. For this, we included the covariates potentially related to treatment and outcome (chronic kidney disease, cardiopulmonary bypass, GPC bacteraemia, heart failure, and shock) in a multivariate logistic model explaining the choice of treatment. The propensity score was built from the selected significant covariates (chronic kidney disease, cardiopulmonary bypass, GPC bacteraemia) and was used in the second model as an adjustment covariate. After matching on the propensity score, we checked that the covariates were statistically balanced between the two treatment groups. Then, we modelled the effect of the treatment on the main endpoint (AKI within first week of treatment) by selecting the covariates associated to this result, with a threshold of significance to 15% in a univariate analysis, and by including in the multivariate logistic model the propensity score as an additional adjustment variable.

The calculations were done under R software [20] by the team EA2415 of the University of Montpellier.

### Results

**Fig. 1:**
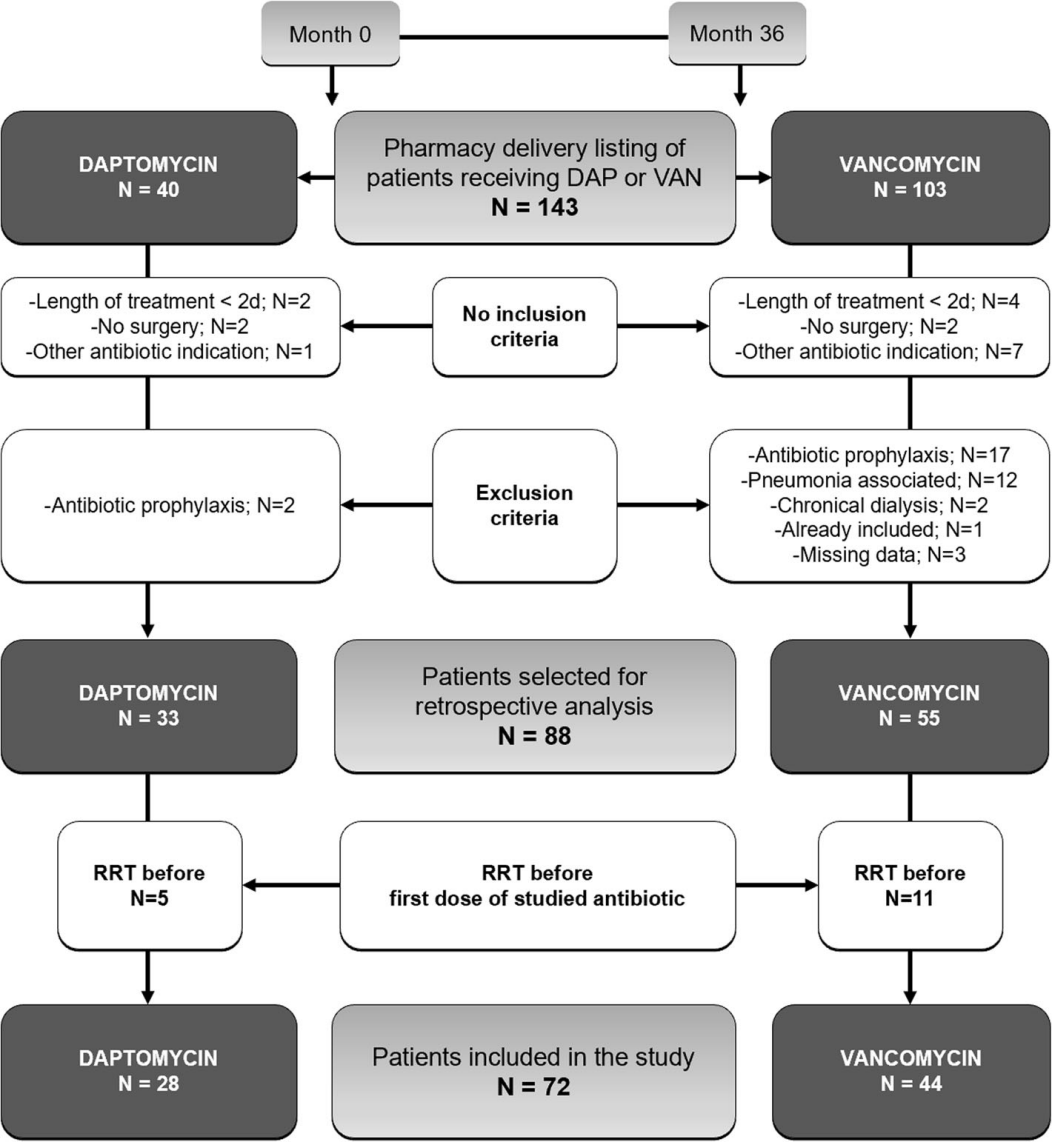
Flow chart of the patient selection process. DAP, daptomycin; VAN, vancomycin; RRT, renal replacement therapy

During the 36-month period of inclusion, among the 143 patients on the pharmacy delivery list for DAP or VAN treatment, 72 patients (28 DAP and 44 VAN) were selected for the study following application of inclusion and exclusion criteria (Fig. 1). After matching to the propensity score, 16 patients of each group were paired. Baseline demographic and clinical characteristics before and after matching are summarized in Table 1. After propensity score matching, characteristics were balanced between the two groups except a significantly higher proportion of patients with heart failure in the DAP group (75% versus 25%, *p* = 0.012).

**Table 1:** Baseline demographic and clinical characteristics of patients before and after propensity score matched analysis

	Before matching			Propensity score matched		
Characteristics	DAP (*N* = 28)	VAN (*N* = 44)	*p*	DAP (*N* = 16)	VAN (*N* = 16)	*p*
Male sex, n (%)	17 (61)	34 (77)	.215	10 (63)	11 (69)	1
Age, y	60.0 [52.3–70.5]	63.0 [54.0–75.3]	.206	56.0 [49.5–69.3]	65.0 [49.0–78.0]	.086
Weight, kg	74.0 [61.5–80.5]	72.0 [64.5–80.0]	.827	71.5 [61.5–79.3]	75.0 [63.0–85.3]	.383
Height, cm	170 [163–173]	167 [165–175]	.821	170 [165–174]	167 [161–169]	.179
History of CRcl < 50 ml/min, n (%)	8 (28.6)	5 (11.4)	.125	2 (12.5)	2 (12.5)	1
History of diabetes mellitus, n (%)	10 (35.7)	11 (25.0)	.478	4 (25.0)	6 (37.5)	.704
History of hypertension	10 (35.7)	23 (52.3)	.258	5 (31.3)	8 (50.0)	.473
Heart failure (EF < 50%), n (%)	18 (64.3)	16 (36.4)	.038	12 (75.0)	4 (25.0)	.012
CPB during surgery, n (%)	9 (32.1)	25 (56.8)	.071	7 (43.8)	7 (43.8)	1
VAD, n (%)	7 (25)	7 (15.9)	.519	5 (31.3)	1 (6.3)	.172
Circulatory shock at baseline, n (%)	8 (28.6)	21 (47.8)	.171	4 (25.0)	9 (56.3)	.149
Others nephrotoxic agents^a^, n (%)	18 (64.3)	29 (65.9)	.910	9 (56.3)	11 (68.7)	.716
SAPS II score at ICU admission	44.5 [35.3–57.8]	45.5 [34.0–57.8]	.894	51.0 [38.5–61.5]	48.0 [35.0–57.0]	.984
SOFA score at baseline	5 [2–9]	6 [3–9]	.388	4 [2–7]	6 [3–8]	.281
Serum creatinine at baseline, μmol/l	110 [77–142]	99 [72–134]	.699	87 [64–126]	94 [69–124]	.440
AKI at baseline, n (%)	7 (25.0)	13 (29.5)	.881	2 (12.5)	6 (37.5)	.220
Previous antibiotic treatment, n (%)	19 (67.8)	24 (54.5)	.381	8 (50)	8 (50)	1
GPC bacteraemia, n (%)	16 (57.1)	12 (27.3)	.022	11 (68.8)	10 (62.5)	.710

Quantitative data are expressed as median [interquartile range]. Baseline is the time of treatment initiation

^a^Within 2 days before or after initiation of treatment

*DAP* daptomycin, *VAN* vancomycin, *CRcl* creatinine clearance, *EF* ejection fraction, *CPB* cardiopulmonary bypass, *VAD* ventricular assist device, *SAPS* Simplified Acute Physiology Score, *SOFA* Sequential Organ Failure Assessment, *AKI* acute kidney injury, *GPC* Gram positive cocci

The reasons for ICU admission (detailed on Table 2) were different between DAP and VAN groups (*p* = 0.004) with more vascular surgery for VAN patients and more cardiologic indications for DAP patients but the rate of cardiac surgery was similar. The primary infectious diseases (reported on Table 2) were distributed slightly differently between groups with more vascular graft infection in the VAN group and more VAD-related infection in the DAP group (*p* = 0.045). Cardiovascular foreign body infection concerned 51% of patients without difference between DAP (*n* = 16; 57%) and VAN (*n* = 21; 48%), *p* = 0.436. A GPC infection was documented in 52 patients (72%), 26 in each group, including different strains of staphylococci isolated in 55% of infections (Table 2).

**Table 2:** Description of medical context and infectious disease

Characteristics	DAP (*N* = 28)	VAN (*N* = 44)	*p*
Reason for ICU admission			.004
Cardiac surgery, n (%)	15 (53.6)	26 (59.1)	–
Vascular surgery, n (%)	2 (7.1)	14 (31.8)	–
Interventional cardiology or short-term mechanical circulatory support, n (%)	9 (32.1)	4 (9.4)	–
Mediastinitis, n (%)	2 (7.1)	0 (0)	–
Primary infectious disease			.045
Infective endocarditis, n (%)	8 (28.6)	15 (34.1)	–
Vascular graft infection, n (%)	2 (7.1)	12 (27.3)	–
VAD infection, n (%)	4 (14.3)	0 (0)	–
Surgical site infection, n (%)	5 (17.9)	6 (13.6)	–
Catheter-related infection, n (%)	5 (17.9)	4 (9.1)	–
Miscellaneous, n (%)	4 (14.3)	7 (15.9)	–
Microbiological identification			.002
MSSA, n (%)	7 (25.0)	4 (9.1)	*–*
MRSA, n (%)	4 (14.3)	3 (6.8)	*–*
CNS, n (%)	13 (46.4)	9 (20.5)	*–*
Enterococcus, n (%)	1 (3.6)	4 (9.1)	*–*
Other Gram-positive cocci, n (%)	1 (3.6)	6 (13.6)	*–*
Other strains or non-documented, n (%)	2 (7.1)	18 (40.1)	*–*

*P*-values for Fisher exact test. *DAP* daptomycin, *VAN* vancomycin, *ICU* intensive care unit, *VAD* ventricular assist device, *MSSA* methicillin-susceptible *Staphylococcus aureus*, *MRSA* methicillin-resistant *Staphylococcus aureus*, *CNS* coagulase negative staphylococci

Daptomycin was administered every 24 h in 24 patients (86%) or every 48 h in 4 patients (14%), with an initial dose of 7.9 [6.6–9.6] mg/kg. Daptomycin doses during treatment course ranged from 7.8 [6.3–9.4] to 8.4 [7.1–9.7] mg/kg. Four DAP patients experienced an asymptomatic increase in creatine kinase, all resolved without consequence or interruption of treatment but by decreasing the dose of daptomycin or increasing the interval of administration. The vancomycin median loading dose in 1 h was 26.7 [18.5–29.7] mg/kg and the continuous intravenous infusion ranged from 21.7 [13.9–26.7] to 29.4 [26.8–33.1] mg/kg daily. In the VAN group, at least one vancomycin serum concentration was below 20 mg/L in 43% of patients or above 30 mg/L in 52%. The treatment duration was longer for DAP than for VAN, with a median of 16 [6–31] versus 8 [4–14] days (*p* = 0.021), respectively.

**Fig. 2:**
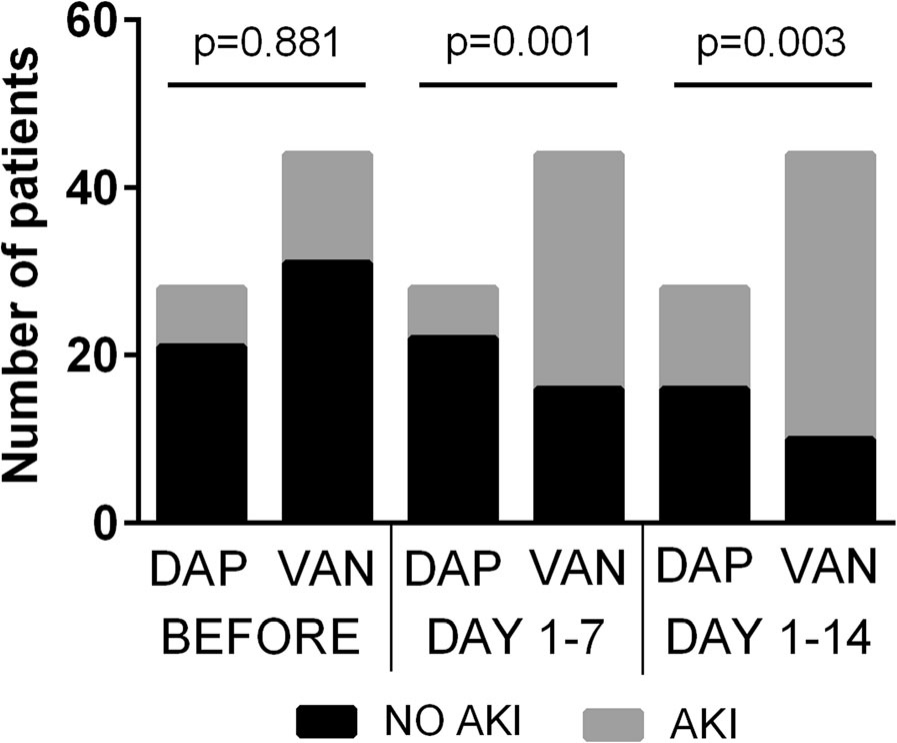
Incidence of acute kidney injury before, between 1 to 7 days and 1 to 14 days after initiation of treatment with daptomycin versus vancomycin (Chi-square test). In black, patients without AKI; in grey, patients with AKI. DAP, daptomycin; VAN, vancomycin; AKI, acute kidney injury

The incidence of AKI at day 7 (primary endpoint) was significantly higher with vancomycin (64%, *n* = 28) than with daptomycin (21.4%, *n* = 6), *p* = 0.001 respectively. Similar results were observed at day 14 (Fig. 2).

Factors associated with 7-day AKI in univariate analysis are reported in Table 3. The multivariate logistic regression model, adjusted to the propensity score (which includes chronic kidney disease, cardiopulmonary bypass and GPC bacteraemia covariates), showed that only vancomycin treatment remained significantly associated with the occurrence of AKI with an Odds Ratio of 4.42 (95%CI: 1.39 to 15.34), *p* = 0.014 (Table 3).

**Table 3:** Covariate factors associated with AKI development in univariate and stepwise multivariate logistic regression analysis adjusted to the propensity score

	Univariable		Stepwise multivariable	
	Odds ratio (95% CI)	*p*	Adjusted-OR (95% CI)	*p*
Male sex	4.22 (1.34–13.28)	.014	–	–
Age	1.03 (0.99–1.07)	.075	–	–
Baseline SOFA score	1.26 (1.09–1.44)	.001	–	–
SAPS II score at ICU admission	1.03 (1.00–1.06)	.043	–	–
Baseline creatinine	1.01 (1.00–1.02)	.038	–	–
Vancomycin treatment	6.42 (2.15–19.12)	.001	4.42 (1.39–15.34)	.014

All variables related to AKI (within 7 days after drug initiation) in univariate analysis, defined by *p* < 0.15 are reported, excepted those resumed in the propensity score. Variables with *p* ≥ 0.15 were not included into the model. All variables entered into the backward stepwise multivariate model including the propensity score were not independently associated with AKI excepted the treatment with vancomycin. *AKI* acute kidney injury, *SOFA* Sepsis-Related Organ Failure Assessment, *SAPS* Simplified Acute Physiology Score

AKI was more severe in VAN group than in DAP patients as assessed by incidence of AKI stage 2 or 3 or GFR decrease of more than 50% and RRT requirement and duration (Table 3).

Clinical failure of initial antibiotic therapy occurred more frequently with vancomycin than with daptomycin, namely in 11 out of 26 VAN patients (42%) and 4 out of 26 DAP patients (15%), with GPC documented infection, *p* = 0.032). Premature treatment discontinuation was not significantly different between DAP and VAN groups (2 (7%) versus 11 (25%), respectively, *p* = 0.055). Several changes were related to vancomycin (11 cases / 44 vs 2 / 28, p = 0.055) including 6 cases of switch from vancomycin to daptomycin. Two failure cases of daptomycin treatment resulted in a switch to vancomycin (occurrence of secondary staphylococci pneumonia, acquisition of resistance).

**Fig. 3:**
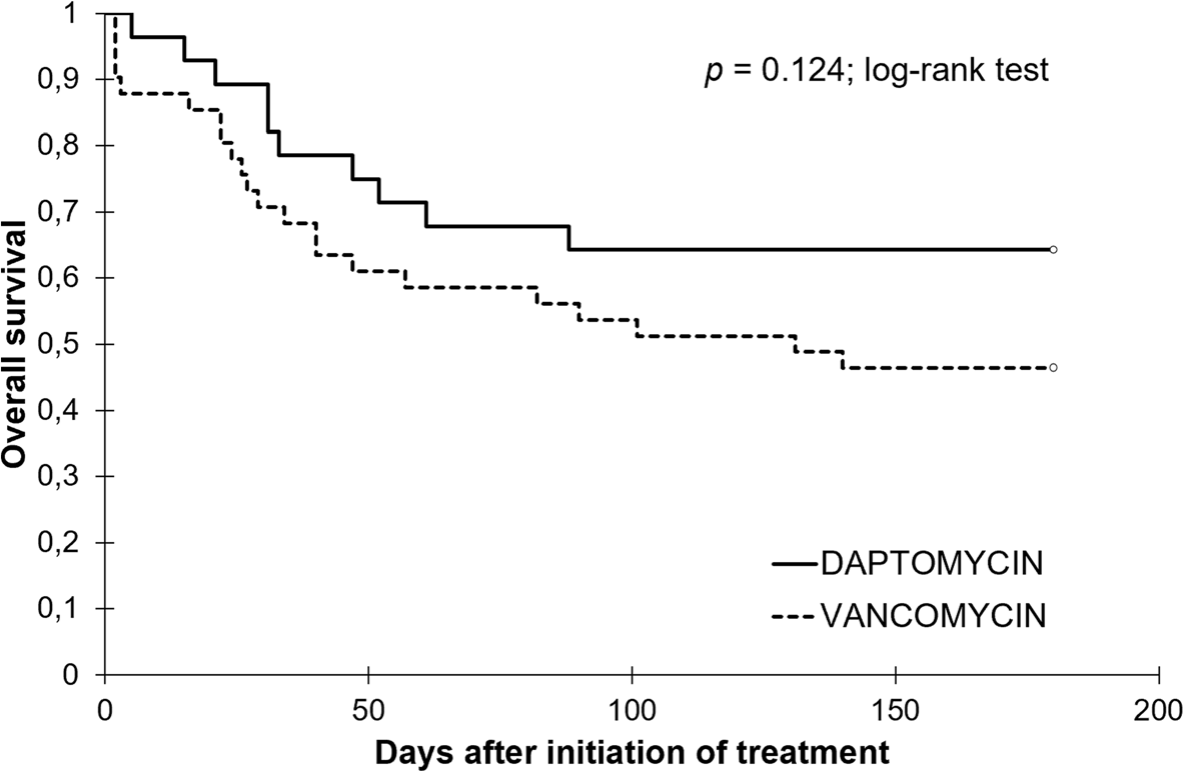
Kaplan Meier analysis of day-180 survival for surgical ICU patients with cardiovascular infection treated by daptomycin (full line) or vancomycin (dotted line)

The ICU and hospital lengths of stay were similar in both groups (Table 4). Mortality was high in this cohort, with no significant difference at month 6 between the VAN and DAP groups (Table 4). The survival curves were not significantly different on a Kaplan Meier analysis (Fig. 3).

**Table 4:** Severity and ICU management of AKI and outcomes

Variables	DAP (*N* = 28)	VAN (*N* = 44)	*p*
AKI severity and management			
AKI stage 2 or 3, or GFR decrease > 50%	7 (25.0)	25 (56.8)	.008
Maximal variation of GFR except RRT (*N* = 57), %	-6 [−46 to + 13]	−31 [−49 to −8]	.055
RRT initiated during treatment, n (%)	2 (7.1)	13 (29.5)	.022
Proportion of ICU days with RRT^a^, %	0 [0–0]	0 [0–13]	.032
Outcomes			
Muscular toxicity (CK > 3xUSL), n (%)	4 (14.3)	0	
Hypersensitivity manifestation, n (%)	0	1 (2)	
Length of ICU stay^a,b^ (*N* = 53), days	9 [4–28]	12 [5–25]	.827
Length of in-hospital stay^a,b^ (*N* = 41), days	47 [19–62]	39 [23–52]	.543
28-day mortality (*N* = 71), n (%)	3 (10.7)	11 (25.6)	.124
180-day mortality (*N* = 69), n (%)	10 (35.7)	22 (53.7)	.142

Quantitative data are expressed as median [interquartile range], except otherwise specified

^a^After study drug initiation

^b^Censored for inpatient mortality

*DAP* daptomycin, *VAN* vancomycin, *GFR* estimate glomerular filtration rate, *RRT* renal replacement therapy, *AKI* acute kidney injury, *CK* creatine kinase, *USL* upper superior limit, *ICU* intensive care unit

## Discussion

Our study compared the impact of antibiotic choice against Gram-positive strains on AKI incidence in ICU patients after cardiovascular surgery, mechanical circulatory support or invasive cardiac procedure. AKI was more frequent and AKI severity higher in patients treated with vancomycin than in patients treated with daptomycin. AKI defined according to KDIGO consensus was 4-fold more likely during the first week of treatment with vancomycin than with daptomycin.

AKI incidence in ICU is about 40%, ranging from 20 to 55% in various large cohort studies [10, 19], with an ICU mortality rate of approximately 40%. Likewise, AKI after cardiovascular surgery, is one of the most common and serious clinical issue, associated with high morbidity and mortality rates [21]. Multiple risk factors and mechanisms have been described such as preoperative renal dysfunction, diabetes mellitus, hypertension, heart failure, anaemia or inflammatory response to CPB [3, 8, 22]. On top of that, nephrotoxic agents may contribute to AKI development, therefore alternative treatments or their discontinuation are strongly recommended whenever it is possible [14].

Our analysis suggests that daptomycin is a less nephrotoxic agent than vancomycin. These results are in agreement with previous observations [23, 24]. In a randomized trial, Fowler et al. found that a renal dysfunction at the end of treatment involved 11% of patients taking daptomycin versus 26% of patients under standard treatment, i.e. low dose gentamicin plus vancomycin or anti-staphylococcal penicillin [17]. The reduction in incidence of AKI observed in Fowler’s study (57%) is very similar to the reduction observed in the present study (67%).

The lower incidence of AKI in daptomycin group than in the vancomycin group was observed despite the use of continuous infusion of vancomycin. Continuous infusion has been reported to be less nephrotoxic than intermittent administration [12, 25, 26]. Nevertheless, the nephrotoxicity of vancomycin remains a serious concern in high risk patients even with continuous administration [18, 27]. In a randomized controlled trial involving 100 high-risk patients, a switch from vancomycin to alternative strategies failed to prevent AKI compared to maintenance of vancomycin (32.7% versus 31.4%) [28]. These results suggest that even short-term initial exposition to vancomycin may be able to impair renal function.

In the present study, observation of RRT exposure in the two groups showed lower indication and shorter duration of RRT in DAP group than in VAN group (Table 4). These results suggest further that daptomycin is less nephrotoxic than vancomycin. Only few studies have addressed the issue of daptomycin or vancomycin treatment during RRT [23].

The VAN group was exposed to numerous overdoses as assessed by serum concentration, in 52% of patients, which may have contributed to the nephrotoxic effect of vancomycin. Inversely, despite the administration of a vancomycin dose consistent with a strict institutional protocol to achieve a target steady-state serum concentration of 20–30 mg/L as recommended [25, 29], low vancomycin levels were also observed (43%) and may have contributed to therapeutic failure. Moreover, untimely discontinuation of initial treatment represented 18.1% of the total population including 6 cases of switch from vancomycin to daptomycin. In patients with non-pulmonary infection due to MR strains (MR *Staphylococcus aureus*, MR coagulase negative staphylococci, *Enterococcus species*), daptomycin given at 6 mg/kg every 24 h is considered equivalent in efficacy compared to standard treatment with vancomycin for some authors [17, 30, 31]. However, in a recent retrospective and matched cohort of patients with MRSA bloodstream infection, clinical failure was significantly higher in the vancomycin cohort than in the daptomycin cohort (45.0% versus 29.0%) [32]. Similar results have been reported in other retrospective studies [24, 33]. These observations highlight the difficulty of achieving a narrow, effective and safe therapeutic target with vancomycin in ICU. Of note, reduced susceptibility to daptomycin may be induced in some strains of *Staphylococcus aureus* by prior vancomycin exposure [30].

Daptomycin was used at high doses as recommended in order to limit the emergence of daptomycin resistance and to speed up bactericidal activity [34, 35]. Drug registry data suggest that doses up to 8 mg/kg every 24 h are well-tolerated and effective [36, 37]. A recently published review concluded that strong experimental evidence suggests that higher doses (e.g. 8–10 mg/kg) of daptomycin should be used for MRSA and enterococcal infections in critically ill patients as well as in bacteraemia and endocarditis [38]. No serum concentration of daptomycin was available, but the treatment regimen seemed appropriate to achieve appropriate antibacterial efficacy, at least not less than vancomycin, with a rather simple protocol, without pharmacological monitoring.

### Limitations

Due to the retrospective design, selection bias and missing data may have influenced the results. The minimal inhibitory concentrations of vancomycin for MRSA strains were not available for most of the patients. Furthermore, no follow up after hospital discharge was organized. The inclusion period was chosen during the first 3 years of daptomycin use in our unit, since we observed an increase in its indication instead of vancomycin in our routine practice after this period. Although the sample of this study is limited and there is always a risk of hidden bias, the absence of a previous or expected randomized trial in this area allows us to retain this result. Despite these limitations, both groups were balanced with respect to baseline characteristics (especially for severity scores SAPS II and SOFA). The striking difference in AKI incidence between both groups, added to the adjustment with the propensity score, suggest these limitations cannot overrule the clinical relevance of the results.

## Conclusions

Vancomycin resulted in significantly more AKI and increased RRT exposure compared to daptomycin in a cardiovascular ICU population. In addition, daptomycin seems well appropriate to maintain an effective and sustained treatment for severe infections, such as endocarditis or cardiac devices and vascular graft infections, specifically in the context of AKI.

## Abbreviations

AKI: Acute kidney injury
CI: Confidence interval
DAP: Daptomycin
GFR: Glomerular filtration rate
GPC: Gram positive cocci
ICU: Intensive care unit
KDIGO: Kidney disease improving global outcome
MR: Methicillin resistance
OR: Odds ratio
RRT: Renal replacement therapy
SAPS II: Simplified acute physiology score II
SOFA: Sequential organ failure assessment
VAN: Vancomycin
